# Supplementary material for: Conditional chemoconnectomics (cCCTomics) as a strategy for efficient and conditional targeting of chemical transmission
Source: eLife. 2024 Apr 30;12:RP91927. doi: 10.7554/eLife.91927 (PMC11060718; doi:10.7554/eLife.91927)
Supplement: Figure 3—source data 3. [file elife-91927-fig3-data3.zip › Figure 3 source data3 part 1.zip!/Figure 3-Source data 3-part1/1067-9-CRISPResso_Report_c33kf3u3.zip!/CRISPResso_on_1067_12/9.Alleles_frequency_table_around_sgRNA_agccaggtgtcgcagacgat.pdf]

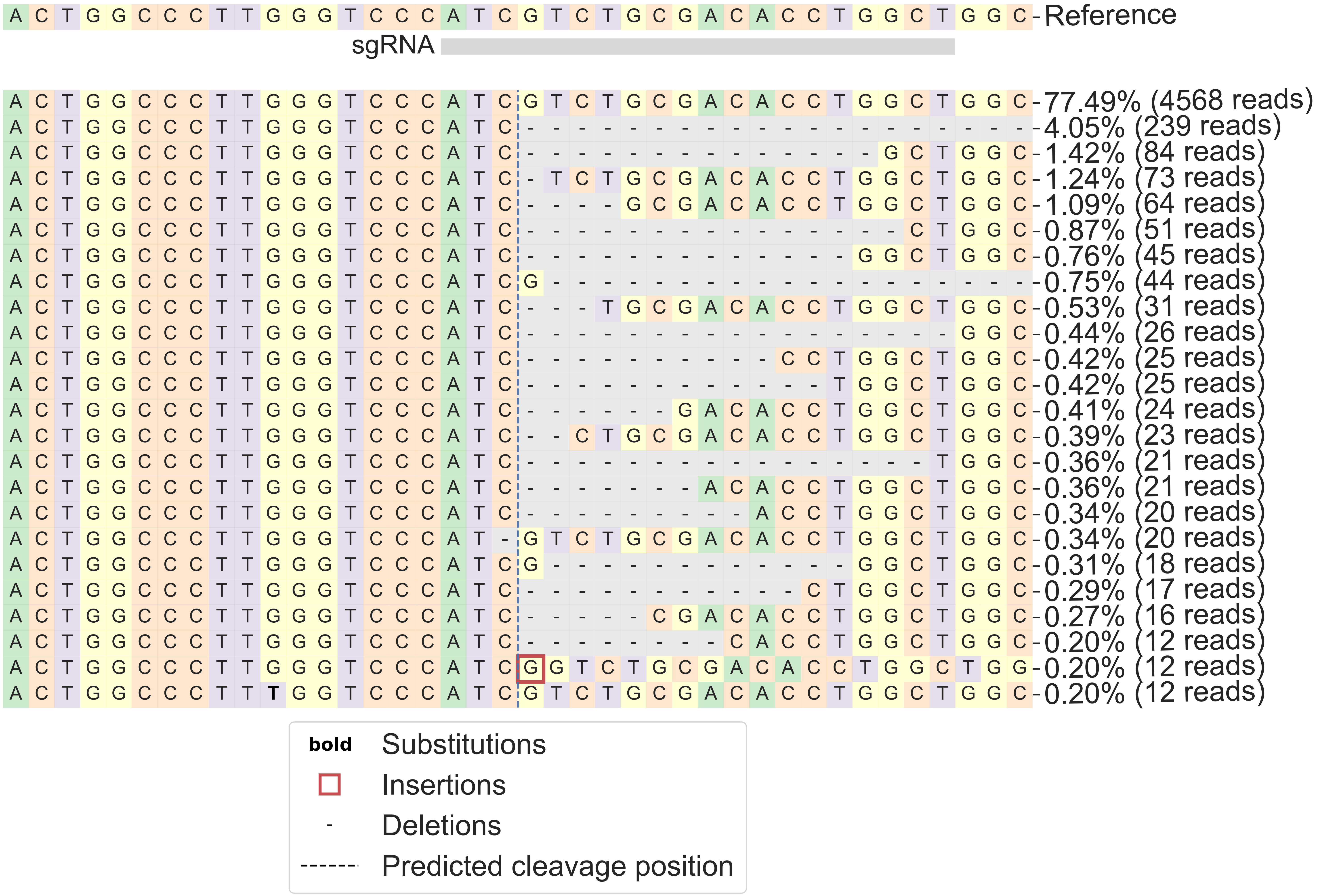
ACTGGCCCTTGGGTCCCATCGTCTGCGACACCTGGCTGGC
Reference
sgRNA
77.49% (4568 reads)
4.05% (239 reads)
1.42% (84 reads)
1.24% (73 reads)
1.09% (64 reads)
0.87% (51 reads)
0.76% (45 reads)
0.75% (44 reads)
0.53% (31 reads)
0.44% (26 reads)
0.42% (25 reads)
0.42% (25 reads)
0.41% (24 reads)
0.39% (23 reads)
0.36% (21 reads)
0.36% (21 reads)
0.34% (20 reads)
0.34% (20 reads)
0.31% (18 reads)
0.29% (17 reads)
0.27% (16 reads)
0.20% (12 reads)
0.20% (12 reads)
0.20% (12 reads)
bold Substitutions
Insertions
Deletions
Predicted cleavage position
